# Irregular optogenetic stimulation waveforms can induce naturalistic patterns of hippocampal spectral activity

**DOI:** 10.1088/1741-2552/ad5407

**Published:** 2024-06-13

**Authors:** Eric R Cole, Thomas E Eggers, David A Weiss, Mark J Connolly, Matthew C Gombolay, Nealen G Laxpati, Robert E Gross

**Affiliations:** 1 Wallace H. Coulter Department of Biomedical Engineering, Georgia Institute of Technology and Emory University, Atlanta, GA 30332, United States of America; 2 Department of Neurosurgery, Emory University School of Medicine, Atlanta, GA 30322, United States of America; 3 Emory National Primate Research Center, Emory University, Atlanta, GA 30329, United States of America; 4 Institute for Robotics and Intelligent Machines, Georgia Institute of Technology, Atlanta, GA 30332, United States of America; 5 Department of Neurosurgery, Robert Wood Johnson Medical School, Rutgers the State University of New Jersey, Newark, NJ 07103, United States of America

**Keywords:** stimulation parameters, theta rhythm, neuromodulation

## Abstract

**Objective.:**

Therapeutic brain stimulation is conventionally delivered using constant-frequency stimulation pulses. Several recent clinical studies have explored how unconventional and irregular temporal stimulation patterns could enable better therapy. However, it is challenging to understand which irregular patterns are most effective for different therapeutic applications given the massively high-dimensional parameter space.

**Approach.:**

Here we applied many irregular stimulation patterns in a single neural circuit to demonstrate how they can enable new dimensions of neural control compared to conventional stimulation, to guide future exploration of novel stimulation patterns in translational settings. We optogenetically excited the septohippocampal circuit with constant-frequency, nested pulse, sinusoidal, and randomized stimulation waveforms, systematically varying their amplitude and frequency parameters.

**Main results.:**

We first found equal entrainment of hippocampal oscillations: all waveforms provided similar gamma-power increase, whereas no parameters increased theta-band power above baseline (despite the mechanistic role of the medial septum in driving hippocampal theta oscillations). We then compared each of the effects of each waveform on high-dimensional multi-band activity states using dimensionality reduction methods. Strikingly, we found that conventional stimulation drove predominantly ‘artificial’ (different from behavioral activity) effects, whereas all irregular waveforms induced activity patterns that more closely resembled behavioral activity.

**Significance.:**

Our findings suggest that irregular stimulation patterns are not useful when the desired mechanism is to suppress or enhance a single frequency band. However, novel stimulation patterns may provide the greatest benefit for neural control applications where entraining a particular mixture of bands (e.g. if they are associated with different symptoms) or behaviorally-relevant activity is desired.

## 1. Introduction

Brain stimulation is a fundamental tool for understanding the mechanisms of neural circuits and treating neurologic diseases such as Parkinson’s disease, epilepsy, and more [1–3]. In most applications, brain stimulation is conventionally delivered using a regular train of electrical pulses at fixed frequency. Recently, studies using irregular temporal waveforms of stimulation have demonstrated potential for increased therapeutic benefit in various neuropsychiatric applications by driving different patterns of neural activity [4]. Following recent successful applications in Parkinson’s disease, device manufacturers have created flexible firmware platforms enabling the design and delivery of arbitrary stimulation waveforms in patients [5–8]. In addition, the improved ability to shape neural activity into desired patterns with optical stimulation methods could improve the causal validity of discoveries in systems neuroscience [9].

Progress in identifying the most effective complex waveform for each application is limited by several factors. First, it is difficult to predict how the brain will respond to different stimulation parameters before directly testing them, in part because it is unknown how complex waveforms differentially control neural activity. Thus, the process of identifying useful parameters remains one of trial-and-error. Conversely, it is intractable to naively try every possible stimulation configuration as there are millions of settings available with modern clinical devices and infinitely many in the space of all possible temporal waveforms [10, 11]. The exploration of irregular parameters in translational settings will benefit from a basic understanding of their effects on brain activity; researchers could combine such knowledge with their understanding of pathological circuit dynamics (spectral biomarkers, neural firing patterns, etc) in various neurological diseases to develop and test new hypotheses in therapeutic and translational settings.

In this study, our goal was to perform a fundamental investigation into the neurophysiological effects of irregular stimulation patterns and characterize how their effects differ from those of standard constant-frequency stimulation, using rodent optogenetic stimulation as a model system. Thus, we asked two questions. First, can irregular stimulation waveforms provide superior entrainment of neural oscillations compared to standard parameters? If so, then such a pattern might hold potential to improve brain stimulation for neurological conditions associated with pathological synchrony. Second, how do the high-dimensional neural activity patterns induced by both irregular waveforms and standard stimulation differ in comparison to behavioral neural activity states? If irregular stimulation patterns can induce behaviorally similar activity, they may hold potential for applications where inducing such neural activity states is beneficial.

To investigate these questions, we used optogenetic stimulation of the medial septum and its effects on hippocampal oscillations as a platform to study the effects of irregular stimulation patterns. Despite limited translational validity, this platform has several suitable features. First, optogenetics (the use of light-activated ion channels to excite or inhibit specific populations of neurons) produces high signal-to-noise ratio stimulation effects that are replicable within and across subjects, are robust to changes over time, and are unobstructed by stimulation artifacts [9]. Second, the medial septum is a well-characterized pacemaker region that robustly controls oscillations throughout the hippocampal formation, providing an effective model to investigate how variability in stimulation parameters affects control of neural activity [12]. In addition, characterizing the effects of medial septum stimulation parameters could improve its potential as a therapeutic target for diseases that impair hippocampal function such as epilepsy, traumatic brain injury, and Alzheimer’s disease [13–15].

In this study, we tested five different classes of optogenetic stimulation waveforms in the medial septum, comprehensively sweeping their amplitude and frequency parameters and measuring their effects on hippocampal activity. First, we characterized the effects of varying the stimulation waveform on oscillatory biomarkers with well-characterized roles in hippocampal function—theta power and low gamma power—and found no or limited differences between the effects of different temporal waveforms on these oscillations. To our knowledge, no prior study has characterized the physiological effects of stimulation parameters *in vivo* at such large scales as we present in this study; beyond the stated hypotheses, our investigation therefore provides a system identification of optogenetic stimulation that could prove broadly useful for future studies of brain control. We then used dimensionality reduction methods to demonstrate that meaningful differences between stimulation waveforms arise when studying their effects across a broader frequency spectrum of hippocampal local field potential (LFP) activity. We compared the induced activity patterns to ‘natural’ neural activity recorded during several behavior paradigms. We found that the activity patterns induced by standard constant-frequency pulsatile stimulation were predominantly artificial (i.e. mostly different from endogenously recorded neural activity) whereas the range of activity patterns induced by all other evaluated parameter spaces aligned more closely with neural activity observed during behavior. These results suggest that novel temporal stimulation patterns could provide benefit for neurological conditions where entraining a mixture of frequencies or desired behaviorally relevant neural states, rather than one biomarker, is a potential therapeutic mechanism.

## 2. Methods

Section 2.1 describes the surgery protocol for rodent optogenetic stimulation and recording. Section 2.2 describes the behavioral task and application of stimulation parameters, and section 2.3 describes data signal processing and feature extraction. Sections 2.4 through 2.7 describe computational methods shown throughout the results.

### 2.1. Surgical protocol

Adult male Sprague-Dawley rats (*N* = 4; mean 5500 stimulation trials per subject) (200–250 g) from Charles River Laboratories (Wilmington, MA, USA) were used for this study. Analyses use a within-subject control over all stimulation trials performed per subject to derive the results. All animals were maintained within a 12 h light/dark cycle vivarium with *ad libitum* access to food and water. All procedures were conducted in accordance with Emory University’s Institute for Animal Care and Use Committee.

For each subject, two surgical procedures under anesthesia (1.5%–4% inhaled isoflurane) were conducted using the same protocol as prior experiments [16]. First, the viral vector AAV5-hSynapsin-Channelrhodopsin2-eYFP was injected into the medial septum at a 20 degree angle to the dorsal-ventral axis (0.40 mm anterior, 2.12 mm lateral at the 20° angle from bregma, 5.80 mm ventral to pia along the rotated axis). A volume of 1.8 *μ*l containing 10^12^ particles ml^−1^ was injected with a rate of 0.35 *μ*l min^−1^ using a pulled-glass pipette attached to a stereotactically mounted injector (Nanoject, Drummond Scientific Co., Broomall, PA, USA). Once the pipette was withdrawn, the scalp was stapled closed, and Meloxicam was administered as an analgesic (3–5 mg kg^−1^).

**Figure 1.**
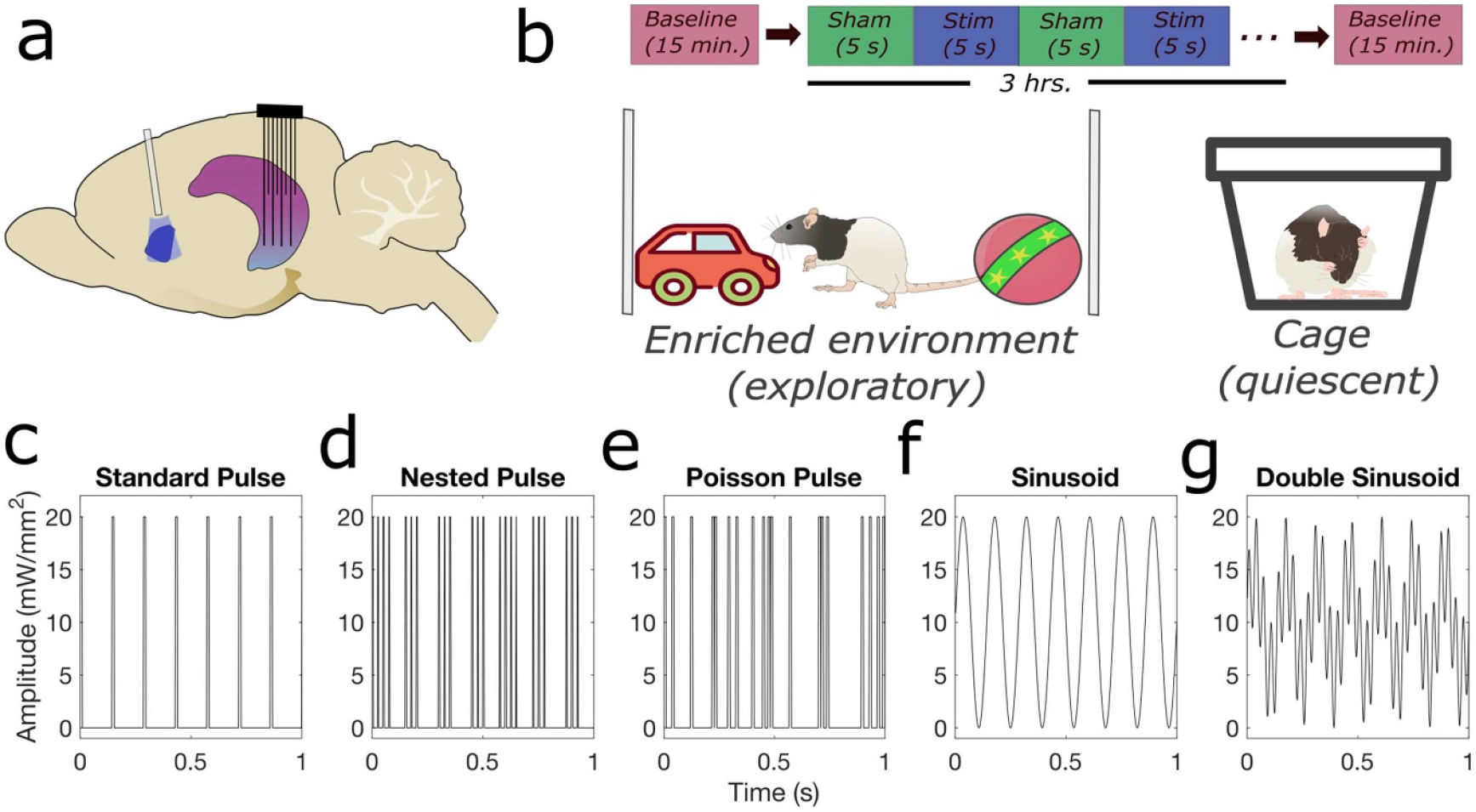
Methods for optogenetic stimulation of the medial septum. (a) Schematic of an implanted optical fiber in the medial septum (blue) and multi-row microelectrode recording from the hippocampus (purple) in the rat brain. (b) Optogenetic stimulation experiments are performed in an open field in an enriched environment promoting exploratory behavior or enclosed cage promoting quiescence (alternating between experiment days). (c)–(g) Depiction of optogenetic stimulation waveforms and parameters. (b) Standard pulse train waveform with parameters: amplitude = 20 mW mm^−2^, frequency = 7 Hz, pulse width = 5 ms. (c) Nested pulse train waveform with parameters: amplitude = 20 mW mm^−2^, train frequency = 7 Hz, pulse frequency = 40 Hz, train width = 80 ms, pulse width = 5 ms. (d) Poisson pulse train waveform with parameters: amplitude = 20 mW mm^−2^, frequency = 7 Hz, pulse width = 5 ms. (e) Sinusoid waveform with parameters: amplitude = 20 mW mm^−2^, frequency = 7 Hz. (f) Double sinusoid waveform with parameters: amplitude = 20 mW mm^−2^, ratio = 0.5, first frequency = 7 Hz, second frequency = 30 Hz.

A second survival surgery was conducted after two weeks, allowing for recovery and optogenetic channel expression. A 16-channel multielectrode array (MEA; Tucker Davis Technologies (TDT), Alachua, FL., USA) with a 1 mm depth offset between rows (designed to target hippocampal CA3 and CA1 pyramidal cell layers) was implanted in the right dorsal hippocampus (centered at 3.50 mm posterior and 2.80 mm lateral to bregma). The MEA was lowered ventrally into the brain until pyramidal single unit activity was observed from both the CA1 and CA3 regions. The ferrule was then driven into the reopened original injection craniectomy at a 20 degree angle to the dorsal-ventral axis to approximately 5.1 mm from the pia along the rotated axis. Correct ferrule depth was determined by applying a 17 Hz, 10 ms, 50 mW mm^−2^ stimulation for 10 s to verify a change in hippocampal activity. Five 2 mm stainless steel screws were mounted on the skull for electrode’s ground and reference wires as well as for the structural support. Finally, the craniotomy was sealed with dental acrylic to secure the electrode and the ferrule in place. Figure 1(a) shows a schematic of fiber and multielectrode array placement.

### 2.2. Stimulation parameter sampling experiments

After a two-week recovery period following the second survival surgery, we applied the following different optogenetic stimulation waveforms and parameters (shown in figure 1) to each subject during awake experiments (described below). Throughout this text, we will use the term ‘waveform’ to denote the class of waveform being used (i.e. standard, sine, nested pulse, or double sinusoid), without regards to any specific parameter combination.

1. Standard pulse train (3D parameter space) (figure 1(c)): pulsatile time series of light defined by an amplitude (10–50 mW mm^−2^), frequency (5–42 Hz), and width (2–10 ms) of rectangular pulses.
2. Nested pulse train (5D parameter space) (figure 1(d)): a time series of rectangular pulses defined by amplitude (10–50 mW mm^−2^), pulse frequency (20–100 Hz) and pulse width (2–5 ms), mediated by a slower on/off rectangular ‘train’ cycle defined by a train frequency (2–12 Hz) and train width (20–80 ms). Note that, for a certain subspace of the parameter space, the parameters may produce either a standard pulse waveform (if the train width is low enough to only fit one pulse in its duration for the given pulse frequency: $\text{TW}>\frac{1}{\text{TF}}$) or other irregularities if the train width and frequency are high enough that adjacent trains overlap in time. During an experiment, any parameter set that met these conditions was discarded and new parameters were sampled before generating and applying the waveform.
3. Poisson pulse train (3D parameter space) (figure 1(e)): time series of rectangular pulses where the time interval between pulses is randomly drawn from a Poisson distribution. Parameters include amplitude (10–50 mW mm^−2^), center frequency of the Poisson distribution (5–42 Hz), and pulse width (2–10 ms). A refractory period was used to ensure that the time between pulses was at least 1 ms.
4. Sinusoid (two-dimensional (2D) parameter space) (figure 1(f)): sinusoidal time series defined by an amplitude (10–50 mW mm^−2^) and frequency (2–50 Hz).
5. Double sinusoid (4D parameter space) (figure 1(g)): a time series of two superimposed sinusoids, defined by the amplitude (10–50 mW mm^−2^) of the overall signal, two sinusoid frequency values (2–50 Hz), and the relative ratio of each sinusoid’s magnitude compared to the total signal amplitude (0–1). The signal is created by generating and summating two sinusoids with relative amplitudes determined by the proportion parameter, both at 0 phase, then scaling the resultant signal such that the minimum value is 0 and maximum value is the amplitude.

The frequency values were chosen based on the time constant of ChR2, which has activation kinetics that saturate at frequencies greater than 50 Hz [17]. Amplitude values were chosen based on previous findings that amplitudes higher than 50 mW mm^−2^ in the medial septum saturate the hippocampal response and produce no greater change in gamma power [18, 19]. Combinations of parameters within these spaces were comprehensively applied during experiments using two sampling strategies: grid search, where parameters are selected according to a predetermined grid; and random search, where all parameter values are drawn from a uniform distribution where the boundaries described above provide the minimum and maximum values. Grid sampling is used to quantify the variability across repeated samples of a single parameter combination, whereas random sampling also allows for interpolation between grid values.

These experiments were performed during two different behavioral conditions: an open-field enriched environment condition (derived from a spatial object recognition memory task) [20] and quiescent behavior (a small, opaque black enclosure) [21]. These two behaviors were chosen to provide two complementary states of hippocampal activity: elevated theta oscillations during spatial navigation and environmental exploration, and depressed theta oscillations during quiescence [21]. The enriched environment consists of a 4′ by 3′ transparent plastic cage, where three large toy objects are placed at different corners of the field to encourage environmental interaction.

The total number of stimulation trials performed was adjusted based on the dimensionality of the parameter space: 2 parameters = 600, 3 parameters = 750, 4 or 5 parameters = 1750 (values are the total number of trials performed per subject over all experimental sessions) [20]. Experiments were performed in approximately 3.5 h blocks, alternating between behavioral paradigms each day until these totals were met for each subject. This long-time duration would naturally incur within-experiment variation in behavior; therefore the goal of each condition was to promote likelihood of the given behavior and ensure that both phenotypes are well represented in the data, rather than guarantee precise behavioral control of neural state. Data are later analyzed based on single-trial differences in underlying neural activity, rather than assuming gross differences between behavioral conditions. To consistently promote exploratory behavior during the enriched environment condition, an experimenter monitored the animal’s interest in the task and briefly paused the experiment for 3 min during periods of low movement to remove the subject, clean the field with ethanol, and replace the objects with different objects.

Individual trials were performed in 10 s blocks, consisting of 5 s for pre-stimulation recording/washout and a 5 s duration waveform at the selected stimulation parameters (a conservative estimate sufficiently long for accurate biomarker measurement based on our prior work). Throughout the experiment, hippocampal LFPs were recorded from the hippocampus using an RZ2 BioAmp Processor and a PZ2 pre-amplifier (Tucker Davis Technologies (TDT), Alachua, FL, USA). Signals were recorded at a sampling rate of 24 414 Hz, and then down-sampled to 2000 Hz before feature extraction and analysis.

### 2.3. Signal processing and feature calculation

For each trial, the power spectral density (PSD) was computed separately for the 5 s pre-stimulation and during-stimulation LFP recordings and averaged across channels (based on our prior studies). The PSD was computed using the Chronux multi-tapered PSD estimate [22] and extracting signal power between 0–100 Hz. Individual power features corresponding to canonical neural bands were extracted by integrating the signal in theta (4–10 Hz) and low gamma (32–50 Hz) ranges.

### 2.4. Modeling effects of parameters on single-band features

#### 2.4.1. Gaussian process (GP) modeling

We used GP regression models to nonlinearly quantify how variability in stimulation parameters impacts spectral activity, rather than assume a simple linear or parametric relationship (using the MATLAB Gaussian Processes for Machine Learning Toolbox v4.2) [23]. Cross validation was performed to choose the optimal kernel configuration for the GP regression. Two mean functions (i.e. constant, affine) and seven covariance functions (i.e. Matern, periodic, rational quadratic, squared exponential, Gabor, linear and polynomial) were tested. Kernels with the lowest normalized mean squared error across the four animals was chosen for each feature, which was the affine/Matern kernel for gamma and theta power. Outliers which were more than three standard deviations from the mean GP were removed and hyperparameters were optimized within the GP framework. These models used the stimulation parameters as inputs and predicted the expected percent increase in power for gamma and theta. Power increase estimates were normalized by the average baseline power (computed by averaging across all pre-stimulation epochs) in each recording/stimulation session, so that the models predicted the relative change in power.

### 2.5. Dimensionality reduction analysis

To derive the neural latent space, the PSD was computed as described above for all 5 s stimulation trials in the range of 0–50 Hz, then log-scaled. This was to ensure that both low and high-frequency regions of the power spectrum were balanced in magnitude and dimensionality, so that the dimensionality reduction methods used did not overweight or neglect one part of the frequency domain when deriving the neural latent space. The resulting dimensionality of the input space was 164 frequency values. We then applied three different dimensionality reduction methods to reduce the post-processed data to a 2D representation.

#### 2.5.1. Uniform manifold approximation and projection (UMAP)

UMAP is a nonlinear manifold learning technique that falls under the class of k-neighbor based graph learning algorithms [24]. Such an algorithm proceeds in two phases: first, the data is used to construct a k-neighbor weighted graph, a network representation where a ‘weight’ is used to quantify the similarity between each data point and its *k* nearest neighbors. Second, a lower-dimensional set of points is created that preserves the structure of this graph (e.g. the weights between each data point and its neighbors are equivalent to those in the original *k*-neighbor weighted graph). Throughout the results, we focus on the latent space derivation provided by UMAP as it is particularly well-suited to balancing the influence of both local and global structure in data (as opposed to emphasizing or overfitting to one of these properties) [25]. The original paper provides a theoretical justification describing how the UMAP algorithm accomplishes this behavior [24].

#### 2.5.2. T-distributed stochastic neighbor embedding (t-SNE)

t-SNE is another nonlinear manifold learning method that falls under the class of *k*-neighbor based graph learning algorithms [26]. This algorithm works by computing pairwise distances between the high-dimensional data points, modeling the set of distances as a probability distribution, then creating an equivalent set of lower-dimensional points that minimizes the Kullback–Leibler divergence between it and the original probability distribution. In practice, t-SNE is thought to primarily preserve local structure rather than global structure present in the high-dimensional data, i.e. clusters of points very close to each other in the latent space would mostly accurately represent distances between these points in the original data, but distances between point clusters that are far apart in the latent space are less likely to reflect the original distance values [27].

#### 2.5.3. Principal component analysis (PCA)

We also performed PCA, which provides a linear method to derive the neural latent space based on its covariance structure [28]. PCA creates orthogonal bases, or linear combinations of input features, which attempt to maximally preserve covariance in the data. This method necessarily captures global structure present in the high-dimensional data, at the cost of discarding nonlinearity and local structure.

### 2.6. Analysis in the neural latent space

Each of the described dimensionality reduction algorithms was used to derive a 2D representation of the high-dimensional data collected during stimulation for each subject. After applying each method, outlier trials were excluded by computing the low-dimensional Euclidean distance between all pairs of points and discarding trials exceeding a mean of three standard deviations of distance away from all other points in the low-dimensional space. To avoid the confounding scenario where low-amplitude stimulation parameters may appear similarly to behavioral trials by simply producing no or little effect on neural activity, all stimulation trials with low-amplitude parameters (less than 30 mW mm^−2^) were excluded before applying the remaining analysis.

#### 2.6.1. Boundary identification

In the neural latent space, we computed boundaries to quantify the area of the neural latent space that was occupied by the data points for each parameter space, describing the subspace of all neural activity patterns that were induced by the given waveform. We first normalized the coordinates of the space such that the minimum and maximum *X* and *Y* coordinates fall within the range [−1,1]. We then defined a discrete grid of 200 × 200 points linearly interpolated between *X* and *Y* values of [−1.1, 1.1] to provide a basis for boundary and area computation. Kernel density estimation was used to fit a 2D nonparametric probability distribution where the probability is proportional to the density of points at a given point in space in each group [29]. We used a 2D symmetric Gaussian kernel with standard deviation of 0.05 and identified boundaries for each group the region of space where *p*(*x,y*) > 0.01, a parameter set that provided suitable boundary fit (e.g. in figures 3(d) and (e)). This boundary identification method was used to identify boundaries for each waveform, data from behavioral recordings, and the total data set collected for a given subject (after outlier exclusion as described above).

#### 2.6.2. Quantifying boundary area

For each region of interest, the normalized area of each region of interest was computed by dividing the area of each individual boundary by the area of the total boundary capturing all data points (after outlier exclusion). The Sorenson-DICE coefficient (a standardized metric in image processing studies) was used to quantify the overlap between different regions of interest as shown in the equation below, where |*X*| and |*Y*| represent the area in pixels of regions *X* and *Y*, respectively [30]

$$\text{DICE}\left(X,Y\right)=\frac{2*\left|X\cap Y\right|}{\left|X\right|+\left|Y\right|}.$$

This metric is equal to 1 when both boundaries are exactly overlapping, 0 when they do not overlap, and is low when the regions overlap partially but have different sizes and/or shapes.

### 2.7. Statistics

Sections 3.1 and 3.2: To quantify differences in bandpower changes between waveform types, we used the non-parametric Kruskal–Wallis test. The sign test was performed to determine difference from zero for each group (*a* = 0.05 as the significance threshold).

Section 3.3: To quantify how the power entrainment effect varied vs. stimulation frequency, we selected the subset of stimulation trials delivered at each discrete frequency grid value and performed a non-parametric one-sample Wilcoxon test to quantify whether there was a significant difference from 0% change. Percent change values were computed for each stimulation trial by summing power in the canonical band that was closest to the stimulation frequency.

Section 3.4: To quantify the properties of spatial representations in the neural latent space, we extend the hierarchical bootstrap test in order to model variability at the level of stimulation trials (rather than individual subjects) [31]. Over five randomizations, we sampled from the neural latent space representation with replacement and recomputed the normalized area and DICE metrics shown in figure 5 to obtain a distribution of the mean value for each waveform. A non-parametric Friedman test is then used to compute a *p*-value for the difference between waveforms while modeling the dependence across subjects. We also report a *p*-value using the Friedman test applied to subject means (i.e. without modeling trial variability).

## 3. Results

**Figure 2.**
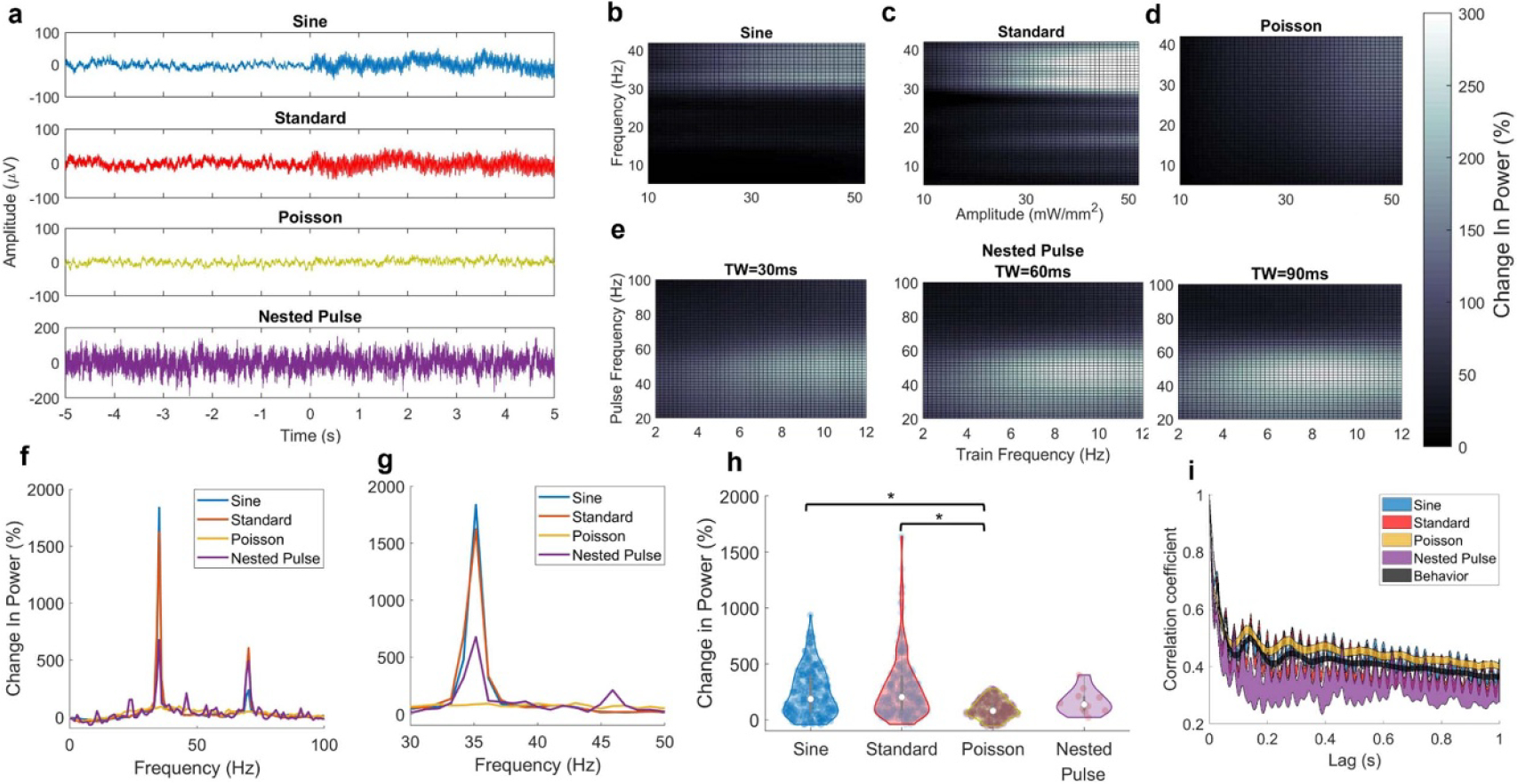
Effect of optogenetic stimulation waveforms on low gamma power. (a) Averaged LFP during 5 s of 35 Hz stimulation parameters for various waveforms (0 s indicates start time of stimulation; −5 through 0 s indicates baseline activity). (b) Mean surface of Gaussian process regression model predicting the low gamma power percent change vs. baseline as a function of standard pulse parameters. (c) Equivalent surface plot for sinusoid parameters. (d) Equivalent surface plot for Poisson pulse parameters. (e) Equivalent surface plots for nested pulse train parameters, corresponding to three values of the train width: left = 30 ms, middle = 60 ms, and right = 90 ms. (f)–(g) Modulation profile showing the bandpower percent change from baseline throughout the power spectral density. Left: 0–100 Hz; right: 30–50 Hz. (h) Change in gamma power relative to baseline for all stimulation waveforms. Sine and standard induced a significantly greater gamma power increase than Poisson (Kruskal–Wallis; *p* = 5 × 10^−13^; *N* = 109, 137, 108, and 7 trials, respectively), with no other significant differences among waveforms. (i) LFP autocorrelation during stimulation for all waveforms at 35 Hz frequency. Behavior: average of all trials with no stimulation aggregated for both behavioral conditions.

**Figure 3.**
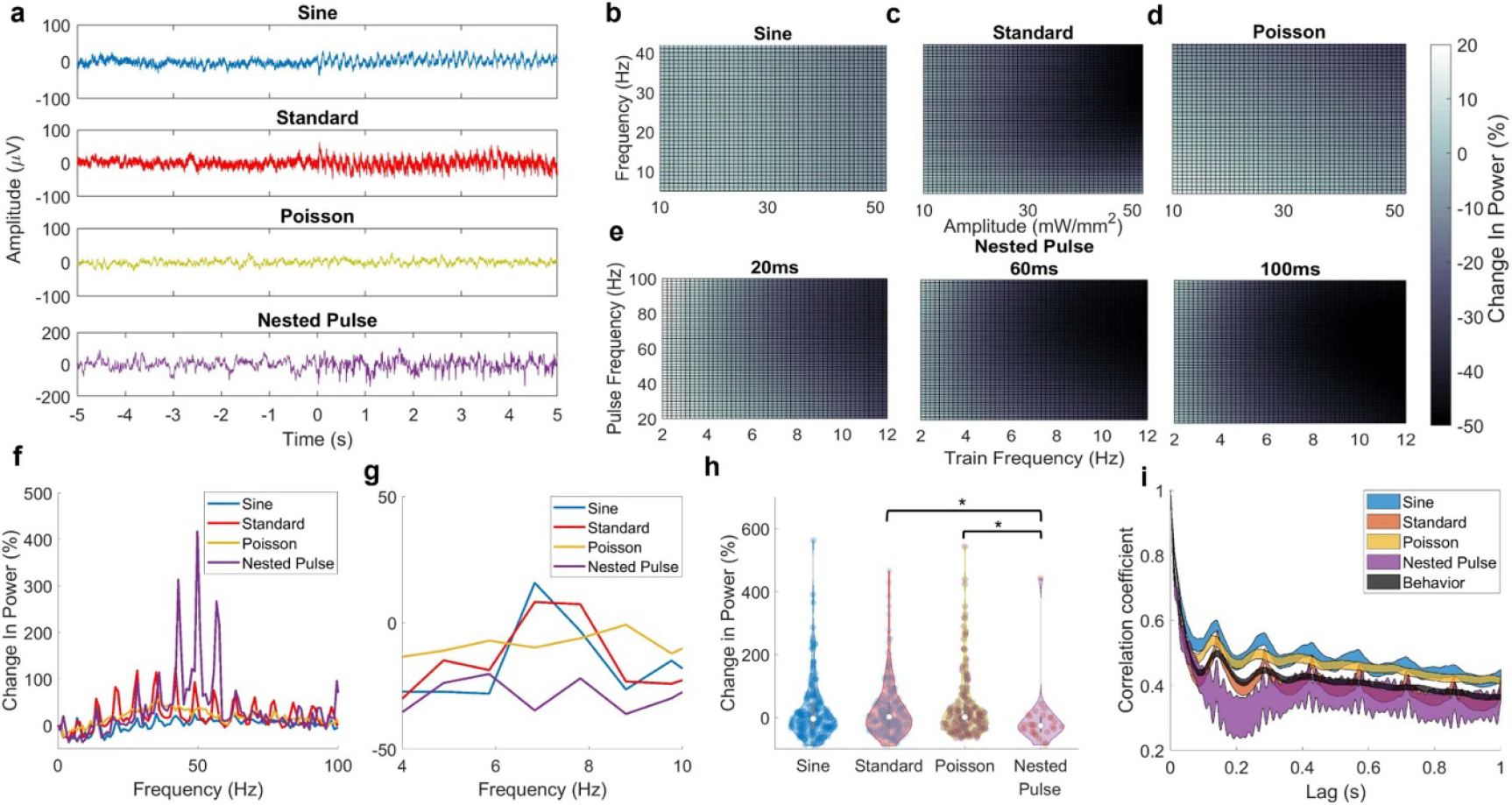
Effect of optogenetic stimulation waveforms on theta power. (a) Averaged LFP during 5 s of 35 Hz stimulation parameters for various waveforms (0 s indicates start time of stimulation; −5 through 0 s indicates baseline activity). (b) Mean surface of Gaussian process regression model predicting the theta power percent change vs. baseline as a function of standard pulse parameters. (c) Equivalent surface plot for sinusoid parameters. (d) Equivalent surface plot for Poisson pulse parameters. (e) Equivalent surface plots for nested pulse train parameters, corresponding to three values of the train width: left = 30 ms, middle = 60 ms, and right = 90 ms. (f)–(g) Modulation profile showing the bandpower percent change from baseline throughout the power spectral density. Left: 0–100 Hz; right: 4–10 Hz. (h) Equivalent frequency and amplitude parameters across waveforms produce no increase in theta power; a significant difference was detected between the nested pulse and standard/Poisson (Kruskal–Wallis; *p* = 0.008; *N* = 113, 130, 110, and 11 trials, respectively), as nested pulse stimulation produced a small decrease in theta power. (i) LFP autocorrelation during stimulation for all waveforms at 7 Hz frequency.

**Figure 4.**
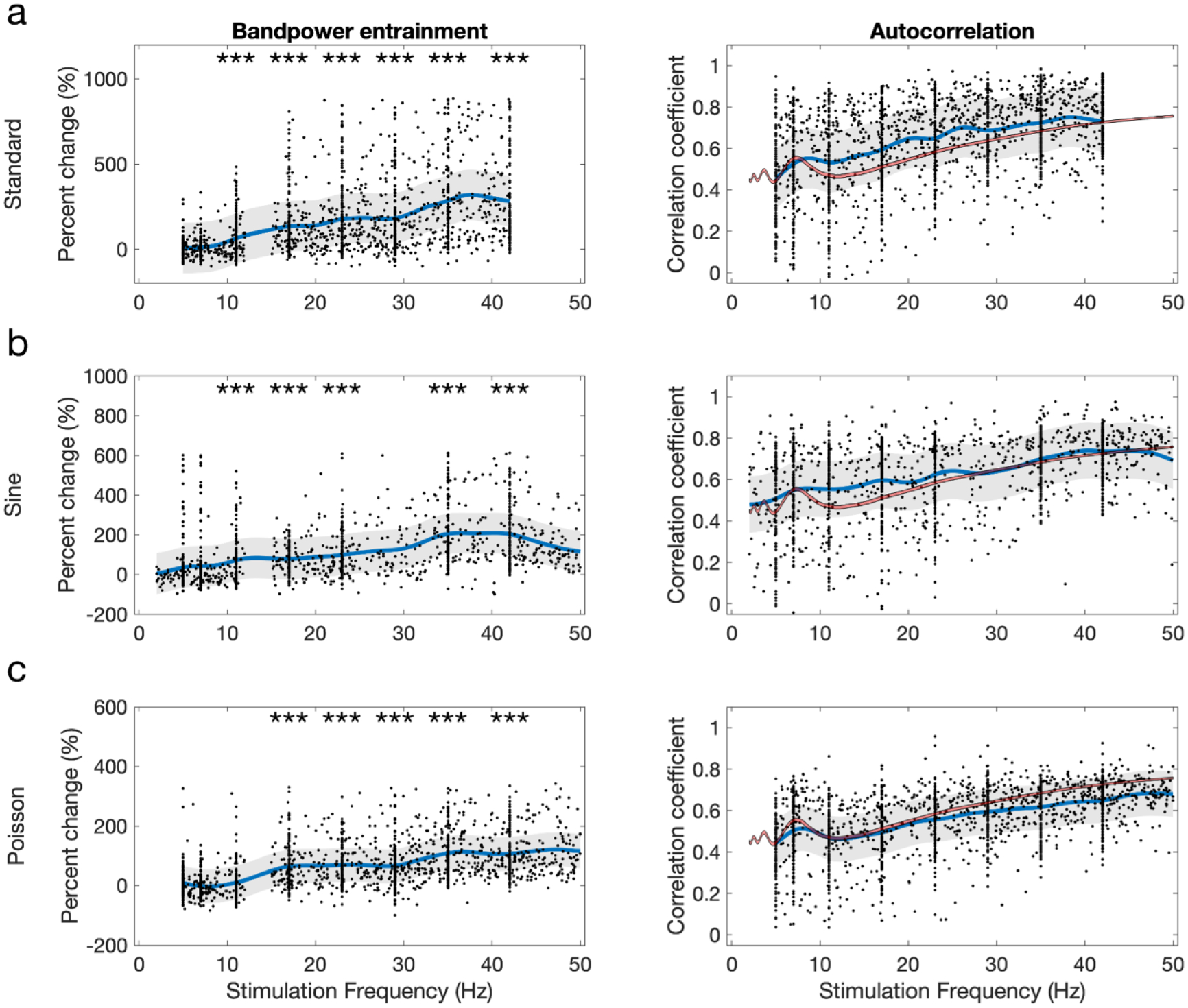
Effectiveness in entraining oscillations vs. stimulation frequency. (a)–(c) Change in power vs. pre-stimulation baseline power (left) and autocorrelation coefficient (right) at the stimulated frequency during stimulation for three parameter spaces (top: standard; middle: sine; bottom: Poisson). For example, the y-value shown at a stimulation frequency of 35 Hz is the percent change in 35 Hz power, or the autocorrelation coefficient of the time series at a 35 Hz time lag. Blue lines and gray shading respectively indicate the mean and standard deviation of a Gaussian process regression model fit to the raw data (black points). Red line: average autocorrelation coefficient during sham trials. No star: *p* > 0.01; *** *p* < 0.0001; Wilcoxon test for median greater than 0. (a) Standard: 5 Hz, *p* = 0.694, *N* = 139; 7 Hz, *p* = 0.185, *N* = 134; 11 Hz, *p* = 2.17 × 10^−9^, *N* = 130; 17 Hz, *p* = 8.15 × 10^−19^, *N* = 132; 23 Hz, *p* = 9.48 × 10^−20^, N = 132; 29 Hz, *p* = 1.56 × 10^−17^, *N* = 135; 35 Hz, *p* = 5.84 × 10^−18^, *N* = 104; 42 Hz, *p* = 1.26 × 10^−22^, *N* = 128. (b) Sine: 5 Hz, *p* = 0.051, *N* = 111; 7 Hz, *p* = 0.065, *N* = 109; 11 Hz, *p* = 2.89 × 10^−7^, *N* = 88; 17 Hz, *p* = 6.02 × 10^−14^, *N* = 97; 23 Hz, *p* = 1.09 × 10^−15^, *N* = 105; 35 Hz, *p* = 3.44 × 10^−17^, *N* = 96; 42 Hz, *p* = 4.32 × 10^−20^, *N* = 112. (c) Poisson: 5 Hz, *p* = 0.032, *N* = 121; 7 Hz, *p* = 0.061, *N* = 114; 11 Hz, *p* = 0.203, *N* = 108; 17 Hz, *p* = 1.57 × 10^−16^, *N* = 116; 23 Hz, *p* = 1.31 × 10^−14^, *N* = 110; 29 Hz, *p* = 1.45 × 10^−13^, *N* = 116; 35 Hz, *p* = 5.72 × 10^−21^, *N* = 118; 42 Hz, *p* = 9,29 × 10^−21^, *N* = 117.

**Figure 5.**
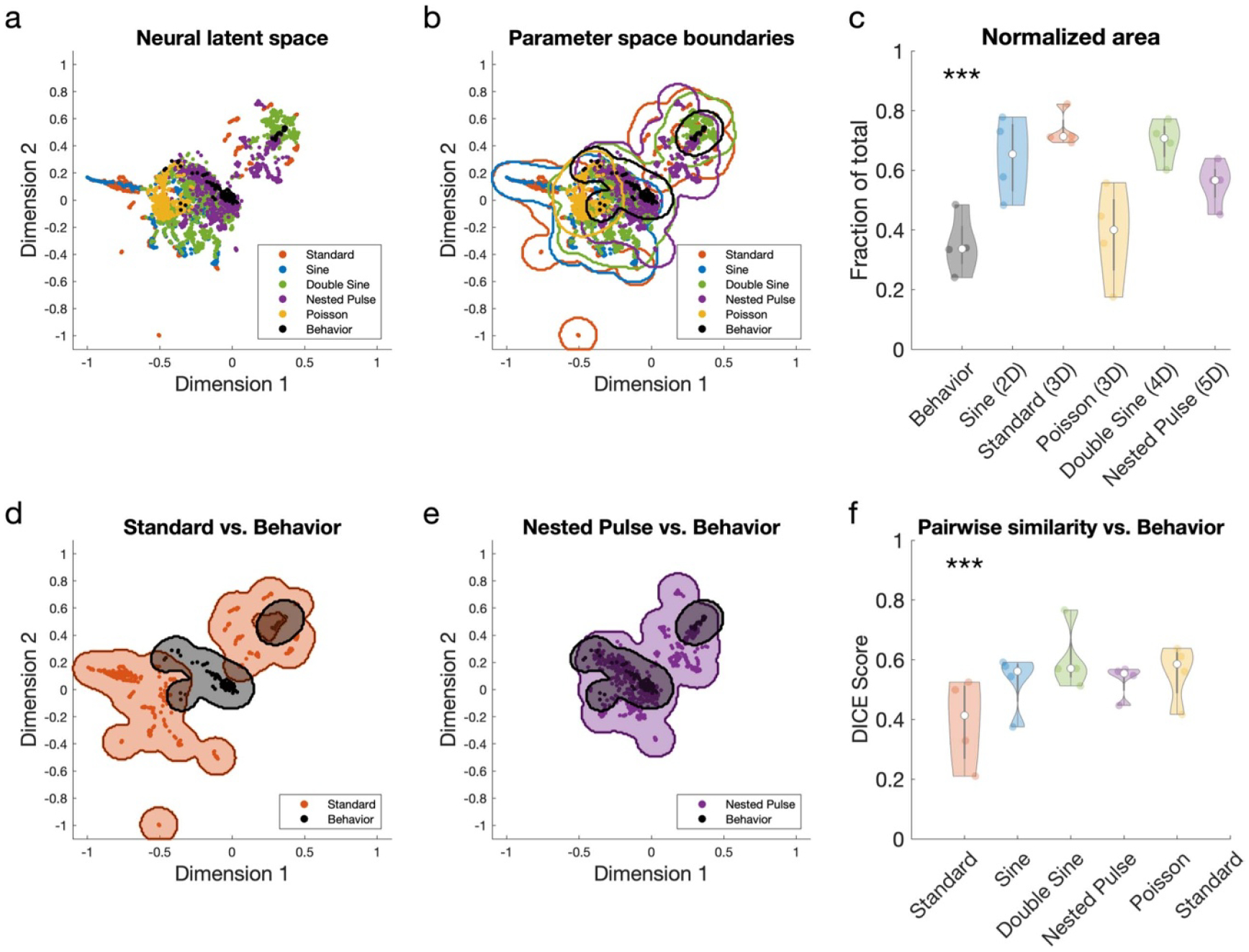
Irregular parameter spaces can induce multi-band activity patterns that more closely resemble behavioral neural activity. (a) Representative neural latent space representation via the UMAP algorithm of PSD during all stimulation parameters tested (example shown for one subject that demonstrated the highest-magnitude power response to optogenetic stimulation). (b) Representative boundaries computed for individual parameter spaces in the UMAP-derived latent space. (c) Normalized area occupied by each parameter space boundary in the latent space (*N* = 4 subjects). The waveforms are ordered by the dimensionality of their parameter spaces, from smallest (*N* = 2D) to greatest (*N* = 5D). (*p* = 5.39 × 10^−17^; bootstrapped Friedman test; d*f* = 5) (d) Comparison of boundaries between standard pulse and behavior parameter spaces for the same subject as in a and b. (e) Comparison of boundaries between nested pulse and behavior parameter spaces. (f) Quantification of the degree to which the boundary of each parameter space overlaps with the boundary of behavior data (*N* = 4 subjects). Low-amplitude stimulation trials (less than 30 mW mm^−2^) were excluded from this analysis. (p = 2.37 × 10^−7^; bootstrapped Friedman test; d*f* = 5).

**Figure 6.**
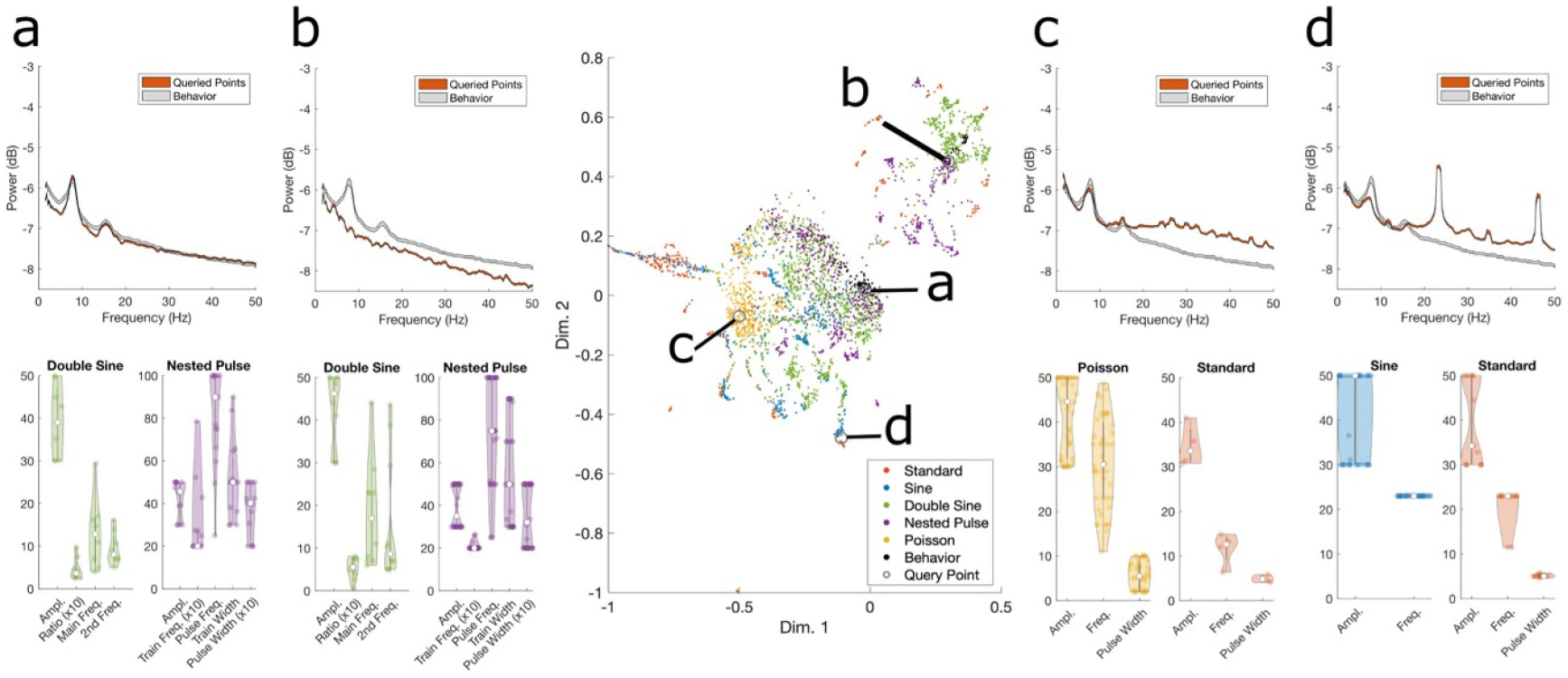
Inspection of the neural latent space. Recreated in the center is a representative plot of the neural latent space for one subject, with four manually selected query points shown as grey circles. For the respective points, each of panels a-d shows the PSD averaged for 50 data points nearest to the manually chosen point, the PSD averaged for all behavioral trials for comparison, and the values of stimulation parameters that were applied during the given trial. The total number of trials associated with each stimulation category is provided here: (a) 26 behavioral trials; 15 nested pulse trials; 8 double sine trials; 1 sine trial. (b) 6 behavioral trials, 10 double sine trials, 34 nested pulse trials. (c) 44 Poisson trials; 4 standard pulse trials; 2 double sine trials. (d) 15 standard pulse trials; 34 sine trials; 1 double sine trial. For double sine parameters, the ‘main frequency’ is the frequency of the sinusoid that had a greater magnitude contribution to the summed waveform (i.e. a ratio parameter greater than 0.5). The values of some parameters are magnified by the factor shown in the *x*-axis labels so that their distributions are more clearly visible.

We systematically varied the parameters of five different optogenetic stimulation waveforms—standard pulse, sinusoidal, Poisson, double sine, and nested pulse stimulation—and measured stimulation’s effects on hippocampal spectral activity during both exploratory and quiescent behavior. We first examined how systematically varying the parameters of each waveform affected two hippocampal oscillations—gamma power (figure 2) and theta power (figure 3), finding that all waveform types enabled equally robust gamma power increase and zero theta power change (with limited exceptions). We then showed that the ability to entrain oscillations was consistent throughout the frequency spectrum for different waveforms (figure 4). Next, we compared the waveforms’ effects in the dimensionality-reduced neural latent space to show that, while standard stimulation could induce a greater range of different neural activity patterns, the induced effects of all other waveforms demonstrated greater similarity to behavioral neural activity states (figure 5). Finally, we inspected the neural latent space to provide insight into the different neural states induced by these waveforms (figure 6).

### 3.1. Parameter effects on hippocampal low gamma power

Figure 2(a) shows the LFP response to stimulation using different stimulation waveforms at an equivalent stimulation frequency of 35 Hz (the double sine parameters were excluded from this stage of analysis due to the lack of a central frequency parameter; trials using a pulse frequency of 35 Hz were used for nested pulse parameters). Stimulation using the sine and standard waveforms demonstrated coherent high-frequency activity that is higher in magnitude than baseline activity and aligned to the onset of stimulation while the nested pulse and Poisson patterns did not. For both the sine and standard pulse spaces (figures 2(b) and (c)), the parameters observed to maximize gamma power corresponded to a maximal optical amplitude (50 mW mm^−2^) and a pulse frequency near 40 Hz, whereas lower frequency and amplitude yielded a decreased power change (consistent with our prior observations using this model) [19, 32]. The Poisson pulse train parameters (figure 2(d)) demonstrated similar gamma-maximizing parameters as observed in the standard pulse space but with a broadening effect of the frequency parameter—a wider range of frequency values was able to produce a lower-amplitude increase in gamma power as compared to the standard pulse space, where a steeper gradient is observed in the regression model surface. In the nested pulse parameter space (figure 2(e)), gamma power remained most sensitive to the optical amplitude and a pulse frequency in the gamma range, with other parameters providing a more modest contribution: a high train frequency was necessary for a maximum response but increasing the train width also increased the gamma response despite lower train frequencies.

In the frequency domain, 35 Hz stimulation was accompanied by a peak in 35 Hz power and a harmonic at 70 Hz which were most prominent for standard and sine parameters. Poisson stimulation produced a broadband increase in power centered at 35 Hz, and nested pulse stimulation produced a less sharp 35 Hz peak with several smaller peaks throughout the power spectrum (figures 2(f) and (g)). We compared the effects of these stimulation patterns by selecting the subset of trials corresponding to 35 Hz frequency and high amplitude (the gamma-maximizing combination for each parameter space): only the Poisson pulse waveform yielded a significantly smaller gamma power increase than other parameters, compared to standard and sine stimulation (Kruskal–Wallis; *p* = 5 × 10^−13^; *N* = 109, 137, 108, and 7 trials, respectively). In an autocorrelation analysis (figure 2(i)), both standard and sine waveforms featured a prominent correlation at 35 Hz which was greatly reduced in the Poisson waveform. The average autocorrelation value across all time lags was higher for Poisson and sine patterns than the other waveforms. Taken together, these results (particularly figure 2(h)) show that the use of novel stimulation waveforms does not provide superior ability to entrain hippocampal gamma oscillations at a particular frequency, but can provide a broader spectral entrainment pattern.

### 3.2. Parameter effects on hippocampal theta power

Figure 3(a) shows averaged LFP recordings for different stimulation waveforms at a 7 Hz stimulation frequency. Consistent with the results for 35 Hz parameters (as shown in section 3.1), the standard and sine waveforms evoked a coherent high-magnitude periodic waveform whereas no change was observed during Poisson stimulation. Nested pulse stimulation produced a response that is salient but less pronounced than was observed with the other waveforms (trials at a train frequency of 7 Hz were used for nested pulse parameters). GP model predictions demonstrate the mean theta power change vs. stimulation parameters for each space (figures 3(b)–(e)). In general, theta power regression models featured a critically lower sensitivity to the effect of stimulation, with large portions of the parameter space producing a near-zero percent change in theta power relative to baseline. The effects of changing stimulation parameters, while apparent, were less pronounced than was observed with gamma power. Sinusoid amplitude and frequency parameters (figure 3(b)) showed negligible sensitivity in the mean response. In the standard pulse space (figure 3(c)), the theta power response was diminished at high amplitudes and frequencies outside of the theta range. Similar to the sine waveform, the theta power response to Poisson pulse (figure 3(d)) parameters was largely insensitive to changes in parameters. In the nested pulse train parameter space (figure 3(e)), a decrease in theta power was observed at higher train frequency values with a limited effect of other parameters.

In the frequency domain, harmonic peaks of 7 Hz were observed for multiple waveforms and were weakest for the sine and Poisson waveforms; nested pulse parameters also produced a strong harmonic associated with the pulse frequency (50 Hz). Stimulation using sine and standard waveforms produced a small increase in power above baseline at exactly 7 Hz but not in the broader range of the theta band (figures 3(f) and (g)). We compared the effects of these waveforms by selecting the subset of trials corresponding to 7 Hz and high amplitude: no waveform was able to consistently increase theta power beyond its baseline value, while the nested pulse train provided a decrease in theta (Sign test, *p* = 0.01). Nested pulse stimulation produced a significantly greater decrease in theta power compared to standard and Poisson stimulation (Kruskal–Wallis, *p* = 0.008, *N* = 113, 130, 110, and 11 trials, respectively). All stimulation patterns displayed a similar 7 Hz autocorrelation profile as was observed in the behavioral data, aside from the nested pulse pattern which featured a prominent 50 Hz oscillatory pattern (associated with the pulse frequency) (figure 3(i)). Standard pulse stimulation displayed sharp peaks at 7 Hz lag spacings which were not observed in the sine and Poisson waveforms. Taken together, these results (particularly figure 3(h)) show that the use of novel stimulation waveforms does not provide superior ability to entrain hippocampal theta oscillations than standard constant-frequency stimulation.

### 3.3. Oscillation entrainment vs. frequency

Based on the findings that stimulation has a different ability to entrain power in low (theta) and high-frequency (gamma) bands, we examined the effect of all stimulation frequencies on power within the stimulated frequency band (figure 4). The magnitude of the power entrainment effect monotonically increased with the stimulation frequency. The lowest frequency value found to produce significant entrainment above baseline power was 11 Hz, and robust power increases were observed above 20 Hz. This trend was consistent across different parameter spaces, but the magnitude of this trend was strongest for the standard pulse parameters and weakest for the Poisson pattern (figure 4(c)), where 17 Hz was the lowest frequency producing significant entrainment. However, the monotonic nature of oscillation entrainment vs. frequency did not substantially deviate for the different stimulation waveforms, aside from the size of the effect. Analysis of the autocor-relational structure in the induced time series showed that the coherence of an oscillation driven at the stimulation frequency did not substantially deviate from the coherence observed at the same frequency during behavior (figures 4(a)–(c))—that is, the influence of frequency on the shape and temporal structure of the induced oscillation also did not change based on the waveform.

### 3.4. Irregular optogenetic stimulation waveforms can induce naturalistic multi-band patterns of activity

We then asked whether stimulation using irregular waveforms can drive different effects on high-dimensional neural activity patterns when compared to standard stimulation. We performed dimensionality reduction on the PSD during all the stimulation trials in this study, in addition to data recorded during quiescent and exploratory behaviors (during which no stimulation was applied). This algorithmic process produced a ‘neural latent space’ representation of the raw PSD data: a 2D set of coordinates where each data point has an equivalent one-to-one match with a data point in the original data set, and the distance relationship between each pair of high-dimensional points is approximately preserved in the 2D representation. Visualization of the neural latent space revealed that individual parameter spaces produce representations that are partially overlapping, but with distinct and separable components (a representative example is shown in figure 5(a); figure S1 shows all representations derived for multiple combinations of subject and reduction algorithm). Notably, the behavioral data organically produced two distinct clusters in the neural latent space, which is consistent with the two behavioral conditions used in the experiment design (visualizable in figures 5(d) and (e)).

Figure 5(b) shows the calculated boundaries for each parameter space (see Methods 2.5.4). For the example shown, boundary contours occupied the following areas: standard = 8006 points (20.0% of the total 200 × 200-point grid); sine = 5579 points (13.95%); double sine = 6937 points (17.34%); nested pulse = 6549 points (16.37%); Poisson = 2020 points (5.05%); behavior = 2776 points (6.94%). Figure 5(c) shows the normalized area which provides a quantification of the ‘potential’ of each parameter space—the range of different neural activity patterns that could be induced by the given set of parameters, compared to the total area of all observed neural activity measurements. All parameter spaces had a greater normalized area than the behavioral data, which had a mean normalized area of 0.28 (*p* = 5.39 × 10^−17^; bootstrapped Friedman test; *N* = 5 replicates, 4 subjects). This result remained significant when performing an underpowered test using only the subject level (Friedman test; *p* = 0.0351; *N* = 1 replicate, 4 subjects). The standard pulse train space displayed the highest normalized area of 0.70, which was greater than the nested pulse train area and Poisson pulse train area. This would mean that, despite their complexity and higher dimensionality, the size of unconventional parameter spaces (double sine, nested pulse train, and Poisson pulse train) did not equivalently correspond to greater flexibility or a larger range of neural activity patterns that can be induced in the neural latent space.

Next, we compared the neural latent space representations produced by these waveforms to neural recordings collected during behavior. Despite occupying a high area, the standard pulse space was found to mainly occupy regions of the neural latent space that are separate from the behavioral region (figure 5(d)), whereas other parameter spaces such as the nested pulse train were found to have greater overlap with the behavioral region (figure 5(e)). When compared to behavioral activity, the standard pulse train space was found to have the smallest boundary overlap, whereas all other parameter spaces featured greater overlap with the behavioral data (*p* = 2.37 × 10^−7^; bootstrapped Friedman test). This result remained significant when testing only at the subject level (Friedman test; *p* = 0.0439; *N* = 1 replicate, 4 subjects). Therefore, the neural effects of standard stimulation were predominantly artificial, whereas all other waveforms had greater potential to drive naturalistic neural activity patterns. This result was consistent across different algorithmic methods (figure S2) and different kernel widths (figure S3) used to derive the neural latent space, and therefore was not a byproduct of the specific machine learning strategy used. To avoid the confound that this effect could be produced by low-amplitude stimulation that simply had no effect on neural activity, we provide three analytical controls: first, all stimulation trials with low-amplitude parameters (less than 30 mW mm^−2^) were excluded from analysis before analyzing the neural latent space; second, we demonstrated robust low-gamma power entrainment consistent with prior studies (figure 2), so the results could not be explained by failure of optogenetic expression; and third, the degree to which the latent space boundaries of different stimulation waveforms were separable was greater for subjects with a higher-magnitude optogenetic response (figure S4).

### 3.5. Inspecting the neural latent space

Finally, we inspected the neural latent space to understand how the effects of these waveforms differed and which specific parameters produced these effects. We manually identified coordinates of interest in the neural latent space, found the 50 nearest neighbors of the selected point, and visualized the mean PSD and stimulation parameters that were associated with the given activity pattern (figure 6). We first chose a coordinate inside one cluster of behavioral activity (figure 6(a)), which showed elevated activity within the theta band and the first harmonic of the theta band. The associated stimulation waveforms were predominantly low train frequency nested pulse parameters and double sine parameters containing a mixture of low (theta) and medium-range (beta) frequencies. Another behavioral cluster (figure 6(b)) showed a lower theta peak with no harmonics, which was again accompanied by double sine and nested pulse parameters at a mixture of low (theta-range) and higher (gamma-range) frequencies. An ‘artificial’ pattern that did not overlap with these clusters (figure 6(c)) showed increased broadband activity centered near 30 Hz, which was mainly produced by Poisson parameters. Another ‘artificial’ pattern (figure 6(d)) showed a pattern with multiple sharp harmonics in the PSD, which was produced by 23 Hz sinusoidal parameters, standard parameters at both 23 Hz and 11 Hz frequencies, and one double sine parameter with beta and gamma-range frequencies. These examples provide insight into the differences between the effects of standard and irregular stimulation: standard pulse stimulation often induced sharp power changes at harmonics of the central stimulation frequency, whereas the mixed-frequency parameters of complex waveforms were able to modulate activity in a given band without the same degree of harmonic entrainment across the spectrum.

## 4. Discussion

In this study, we characterized the neurophysiological effects of irregular optogenetic stimulation waveforms and compared their effects to those of standard constant-frequency stimulation. We first found that stimulation using irregular waveforms did not provide any superior ability to entrain neural oscillations vs. constant-frequency stimulation, as shown by their equivalent ability to modulate gamma power (figure 2), lack of ability to entrain theta power above baseline (figure 3), and similar entrainment effects when varying the stimulation frequency (figure 4). Second, we showed that meaningful differences between stimulation waveforms arise when studying their effects on hippocampal activity in the neural latent space and that the overall activity patterns induced by standard constant-frequency pulsatile stimulation are predominantly artificial (i.e. most different from endogenously recorded neural activity). In contrast, the range of activity patterns induced by all other evaluated parameter spaces aligned more closely with activity observed during behavior (figures 5 and 6). These findings suggest that irregular stimulation patterns are not useful in control applications where the desired mechanism is to suppress or enhance a particular oscillatory frequency band but may be most beneficial in settings where inducing a desired relationship between multiple power bands or driving complex behaviorally-relevant brain states could be effective. Below, we discuss: (1) the implications of these findings for informing future brain stimulation studies, (2) limitations, including relevance to electrical brain stimulation and behavior, (3) potential mechanisms underlying the observed differences between waveform effects, and (4) implications for designing subject-specific parameter optimization approaches.

When investigating the effects of stimulation using each waveform on hippocampal oscillations, we found several counter-intuitive results: pan-neuronal optogenetic stimulation of the medial septum was unable to increase low-frequency activity above its baseline value across all parameters tested. Further, varying the temporal pattern of stimulation did not produce a substantial difference in the entrainment of either spectral biomarker. The former observation is particularly surprising given that the medial septum is known to be a predominant driver of the hippocampal theta rhythm [12, 13]. Taken together, these results contradict common conventions in translational neuromodulation studies. For example, a study investigating stimulation in the medial septum as a treatment for epilepsy might choose theta-frequency or theta-burst stimulation based on literature showing that hippocampal theta rhythms are seizure-resistant, then measure its effect on seizure frequency compared to sham stimulation, based on a mechanistic hypothesis of entraining hippocampal theta rhythms [33, 34]. Our results suggest that the fundamental mechanistic premise behind such a study may be incorrect and could easily go untested due to the experimental challenges of measuring neural activity during electrical stimulation and evaluating changes in behavioral symptoms. Ultimately, because the effects of brain stimulation can be counter-intuitive and vary nonlinearly with the brain region and modality being studied, translational neuromodulation studies would benefit from empirically measuring how multiple candidate stimulation patterns impact neural activity to assert that underlying mechanistic assumptions are true before evaluating their therapeutic effect.

It is important to acknowledge that the results of this study may not generalize to therapeutic applications of electrical brain stimulation, as electrical and optogenetic stimulation influence neural activity through different mechanisms. For example, electrical stimulation activates non-neuronal tissue and produces different activation thresholds for different neuronal elements (e.g. axons, axon terminals, and dendrites) [35]. In contrast, optogenetic activation of ion channels is more uniform and features a different spatial profile that is influenced by the spread of light and viral expression in neural tissue [36]. In addition, computational studies suggest that neural circuits may respond to these stimulation types with a different degree of state-dependence and dynamics [37]. However, in certain circumstances their effects might be similar: when engineered to respond to similar frequencies and target the appropriate circuit elements, optogenetic activation can mimic the therapeutic effects of electrical stimulation in a Parkinsonian rodent model [38] and produce similar synaptic responses to individual stimulation pulses (though, with differences for sustained pulse bursts) [39].

In general, there are not many studies quantitatively comparing the two stimulation modalities outside of computational models, or even performing general system identification of optogenetic stimulation responses. Future studies performing such research in different brain regions and with different modalities might reveal whether our study of stimulation waveforms generalizes to new experimental settings. Until then, these results should be considered primarily relevant to pan-neuronal medial septum optogenetic excitation.

In one key limitation, our study did not perform the necessary controls to assess whether stimulating to induce a given neural pattern (e.g. elevated theta) would causally produce the behavior associated with that neural activity state. It is not necessarily likely that this hypothesis would hold true, for several reasons. First, we measured from only one brain region due to its responsiveness to septal stimulation; however, the neural correlates of memory and spatial navigation critically involve other areas that interact with the hippocampus [40], which may respond differently (or not at all) to septal stimulation. In addition, differences may arise between stimulated and behavioral activity when modeling hippocampal activity at higher resolution, e.g. with single-unit firing patterns or LFP phase dynamics, as demonstrated by recent studies of rhythmic sensory stimulation [41, 42]. Nevertheless, the analytical framework and results we demonstrate could inform future studies further investigating the effects of irregular stimulation with more spatiotemporally resolute stimulation and recording modalities.

One prominent limiting factor in our study was trial-to-trial variability across repeated stimulation events. For example, even when modest significant differences arose between waveforms, the difference in means was negligible compared to the variability of applying a single parameter combination across different trials (figures 2(h) and 3(h)). This variability may result from multiple sources of state-dependence in the effects of stimulation [43]; the effects of stimulation nonlinearly summate with the animal’s natural neural activity state which varies throughout the experiment, and the modulation effect of stimulation itself at various frequencies can change nonlinearly based on the animal’s behavioral state [21, 44, 45]. In this study we used open-loop stimulation as a model for how brain stimulation is typically performed clinically, as there are major technical barriers to achieving precision state-dependent control of neural systems in patient care [46]. How the observed effects of stimulation parameters vary with brain state is a complex topic meriting its own dedicated study. Future work in this effort could explore how a dimensionality reduction approach may be augmented to identify the neural state components that predict variance in the effect of stimulation and achieve more precise modeling of stimulation effects.

In this study, we demonstrate a feasible methodology using dimensionality reduction that can be used to quantify differences in the effects of stimulation parameters at scale. The extension of these methods to challenging electrical stimulation domains may be aided by recent well-validated advancements in artifact rejection [47] or by measuring evoked potentials observed in-between stimulation pulses [48, 49]. The specific dimensionality reduction strategy could also be augmented by using novel methods that are targeted towards specific types of structure in neural data, including calcium imaging [50, 51] or the dynamics of neural spiking [52]. Such approaches may also be combined with novel data-driven optimization algorithms to search for subject-specific stimulation patterns that maximize the similarity between the stimulation-induced and desired brain state [53]. Our work could supplement the use of genetic algorithms and biophysically informed computational models of neural systems, which can suggest many more candidate stimulation patterns for empirical testing but suffer from limited ecological validity [54]. To resolve challenges with state-dependence (as described above), the integration of data-driven latent space modeling and millisecond-timescale feedback control may the most robust approach to precisely drive desired activity patterns in neural circuits to causally interrogate their function and treat disease [55].

We showed that conventional pulsatile stimulation induces patterns of neural activity that were most different from behavioral activity. There are two specific features unique to standard pulse stimulation that may explain the artificiality of the corresponding effects: the abrupt rising edge of rectangular stimulation pulses and the constant-frequency nature of the waveform. All other waveforms examined in this study differed by either smoothly changing the amplitude over time (sinusoid and double sinusoid) and/or distributing the frequency content of the waveform into a mixture of slow and fast frequencies (nested pulse train and Poisson). Waveforms with these factors suppressed the high-magnitude harmonics of the stimulating frequency that were observed with standard pulse stimulation. Rectangular pulses might drive artificial neural responses by promoting highly synchronous firing across the spatial volume of tissue activated at the rising edge of the stimulation pulse, whereas a smooth increase in the amplitude of stimulation would result in different neuronal populations firing at different times within the spatial field of light. Future work may investigate this hypothesis by examining the timing of neuronal population responses across a microelectrode array during pulsatile vs. sinusoidal stimulation and examine whether the biomimetic utility of sinusoidal stimulation can be replicated with electrical stimulation by smoothly adjusting the amplitude of an electrical pulse train [56].

**Figure 7.**
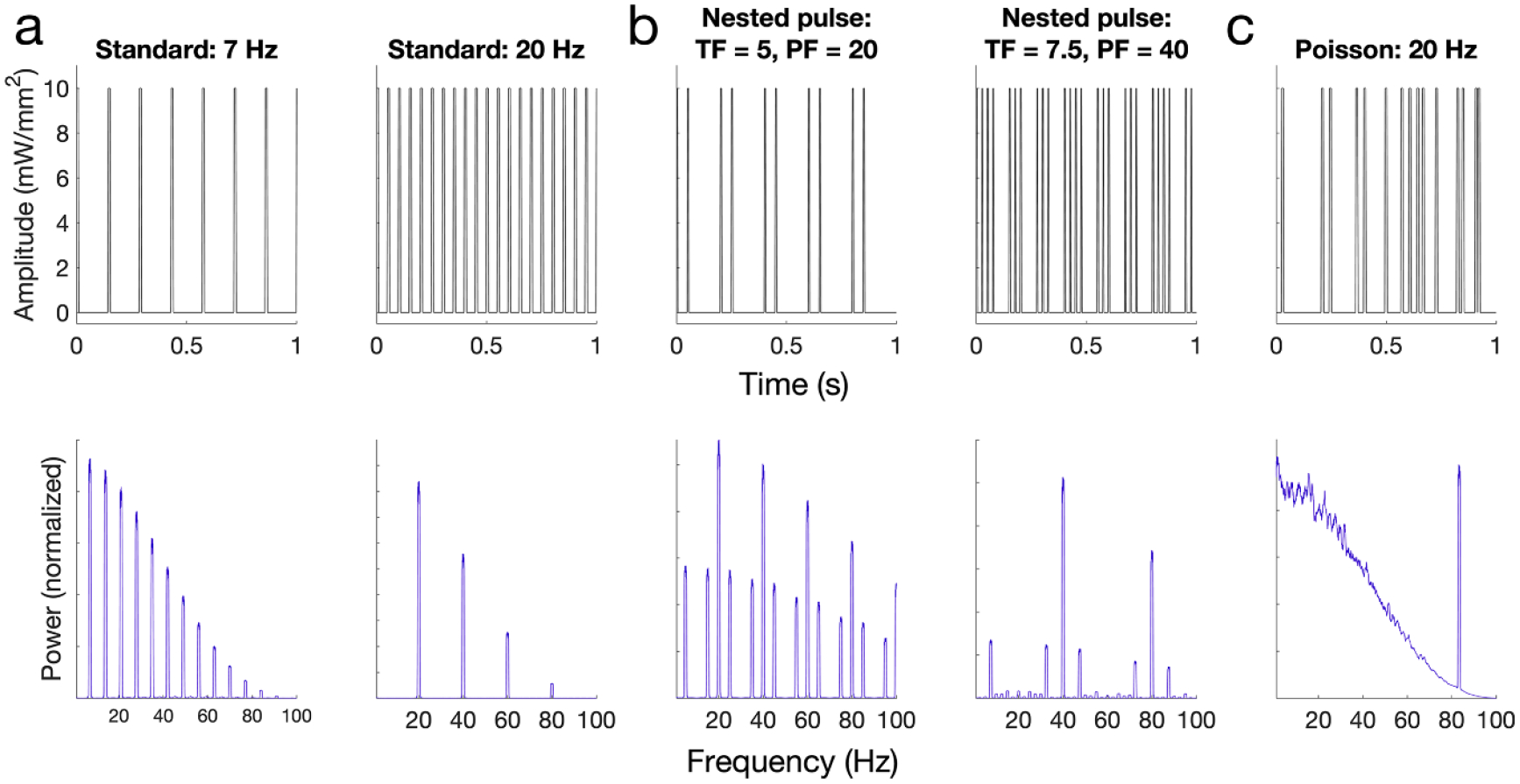
Stimulation waveforms and their spectral content. Each column shows the time-domain (top) and frequency-domain (bottom) PSD representations of standard pulse (a), nested pulse (b), and Poisson pulse (c) waveforms at various parameters. (a) The standard pulse train features spectral components at multiple harmonics of the pulse frequency, with a similar power fall-off vs. frequency. (b) Nested pulse train (NPT) waveforms differing in train frequency (TF) and pulse frequency (PF), with other parameters held constant: train width = 80 ms and pulse width = 5 ms. The nested pulse waveform features the largest spectral components at the pulse frequency and its harmonics, with smaller peaks surrounding each harmonic peak at pulse frequency ± train frequency. (c) The Poisson waveform PSD was computed by generating 1000 independent Poisson waveforms at the given frequency, then separately computing and averaging their PSD representations. The Poisson waveform features a broadband power spectral density profile, with a faintly visible peak at the stimulation frequency and a large peak near 80 Hz resulting from the 2 ms minimum refractory period between pulses. Sine and double sine waveforms are not shown, as the definition of their spectral content is trivial (one or two peaks at the sinusoidal frequencies).

It is important to note that spectral content of the waveform may strongly influence the resulting neural response, especially with respect to the harmonic effects of stimulation. In figure 7, we show examples of the PSD of the stimulation waveform itself for several standard, nested pulse, and Poisson parameter combinations. In particular, the neural effects of standard and nested pulse stimulation demonstrated key similarities to their spectral content. The nested pulse waveform features a characteristic harmonic pattern with three peaks at the pulse frequency ± the train frequency (figure 7(b)), which appears in the hippocampal PSD (figure 3(f)). Additionally, the hippocampal PSD features harmonic peaks at multiples of the pulse frequency during standard stimulation (figure 3(f)), replicating its spectral pattern (figure 7(a)). However, there were several differences: harmonic peaks remained in the hippocampal LFP during sine simulation (albeit with lower magnitude), despite this waveform containing zero harmonic power. The spectral components of each waveform were also dampened in the low-frequency range, consistent with the results shown in figure 4. These findings suggest that, in the right circumstances, designing stimulation waveforms in the frequency domain may be a simple and effective way to achieve desired effects on neural activity.

We found that harmonic entrainment of frequencies outside the stimulated frequency band was a key contributor to the artificial effects of standard pulse stimulation, but the physiological mechanisms of these harmonics remain incompletely understood. Various computational modeling results provide evidence that the recurrently connected structure of neuronal networks can give rise to both superharmonic and subharmonic entrainment effects when driven by external stimuli [57, 58]. However, in experimental results these effects may partially or fully arise as an artifact of standardized Fourier transform-based analysis methods, which can discover increased harmonic power in signals that simply deviate from a perfectly sinusoidal waveform shape (but do not have a true physiological oscillation at the harmonic of the central frequency) [59]. Studying such harmonics in experimental recordings may be aided by analysis methods which have been developed to suppress such harmonics for the study of cross-frequency coupling in neural systems [60]. A common neural control objective may be to drive a central neural oscillation without inducing harmonics in other oscillations which could be harmful; for example, Parkinsonian dyskinesia is associated with power in a subharmonic band of clinically standard DBS frequencies [61, 62], and prior studies of DBS in epilepsy have been designed to avoid harmonics in frequencies that are expected to induce seizures [63]. We observed that the Poisson waveform, when compared to standard pulse stimulation at an equivalent central frequency, increases power at the central frequency while obscuring the effects of harmonics. These empirical observations support recent theoretical work showing that adding noise to the inter-pulse interval of a constant-frequency stimulation waveform (‘dithering’) can suppress harmonic stimulation effects [64]. There may be a quantitative tradeoff between these two properties: having low inter-pulse noise produces greater entrainment at the central frequency, but at the cost of increased harmonics; higher inter-pulse noise might lead to reduced harmonics, but also decreased entrainment at the central frequency. We defined the randomized inter-pulse interval using the Poisson distribution, creating a very wide spectral profile for both the stimulation waveform itself (figure 7(c)) and its neural effect. Future studies might define noise around the central stimulation frequency using a narrower distribution where this noise value can be directly parameterized (e.g. Gaussian) to control this tradeoff more precisely.

We observed that the higher-dimensional parameter spaces associated with the nested pulse and double sine waveforms did not produce a larger representation in the neural latent space than standard stimulation, let alone one that was quantitatively consistent with their increased dimensionality (figure 5(c))—a potentially important result for the subject-specific optimization of stimulation parameters. Whether for translational or clinical applications, subject-specific stimulation parameters are typically identified by first defining a broad parameter space, then iterating through regions of the space manually or algorithmically to identify an optimum [11, 65]. Such increased size comes at a cost, as the addition of each new parameter results in an exponential increase in the amount of data needed to understand and model the effects of stimulation or traverse the parameter space to find effective stimulation settings for a given application or subject [10]. Much of this added dimensionality may be redundant, i.e. correspond to similar effects on brain activity (as seen with the small latent representation of the high-dimensional nested pulse parameter space in figure 5(c)). This ‘combinatorial explosion’ effect is seen in the development of commercial deep brain stimulation devices, which have grown in complexity from thousands to millions of total parameter combinations available over years of clinical application [66]. Our results suggest that adding even a simple dimension of temporal irregularity (e.g. randomized inter-pulse interval) may be a more effective and complementary strategy to enable new control capabilities when defining a parameter space for optimization, while limiting the added redundancy that can make navigating the parameter space less tractable.

## Supplementary Material

Supplementary material

Supplementary material for this article is available online

## Data Availability

The data that support the findings of this study are openly available at the following URL/DOI: https://github.com/ericrcole/opto_param_analysis.
