# Supplementary material for: Irregular optogenetic stimulation waveforms can induce naturalistic patterns of hippocampal spectral activity
Source: J Neural Eng. Author manuscript; Available in PMC 2025 Jul 1. (PMC12212926; doi:10.1088/1741-2552/ad5407)
Supplement: Supplementary material [file NIHMS2089799-supplement-Supplementary_material.pdf]

SUPPLEMENTARY MATERIAL

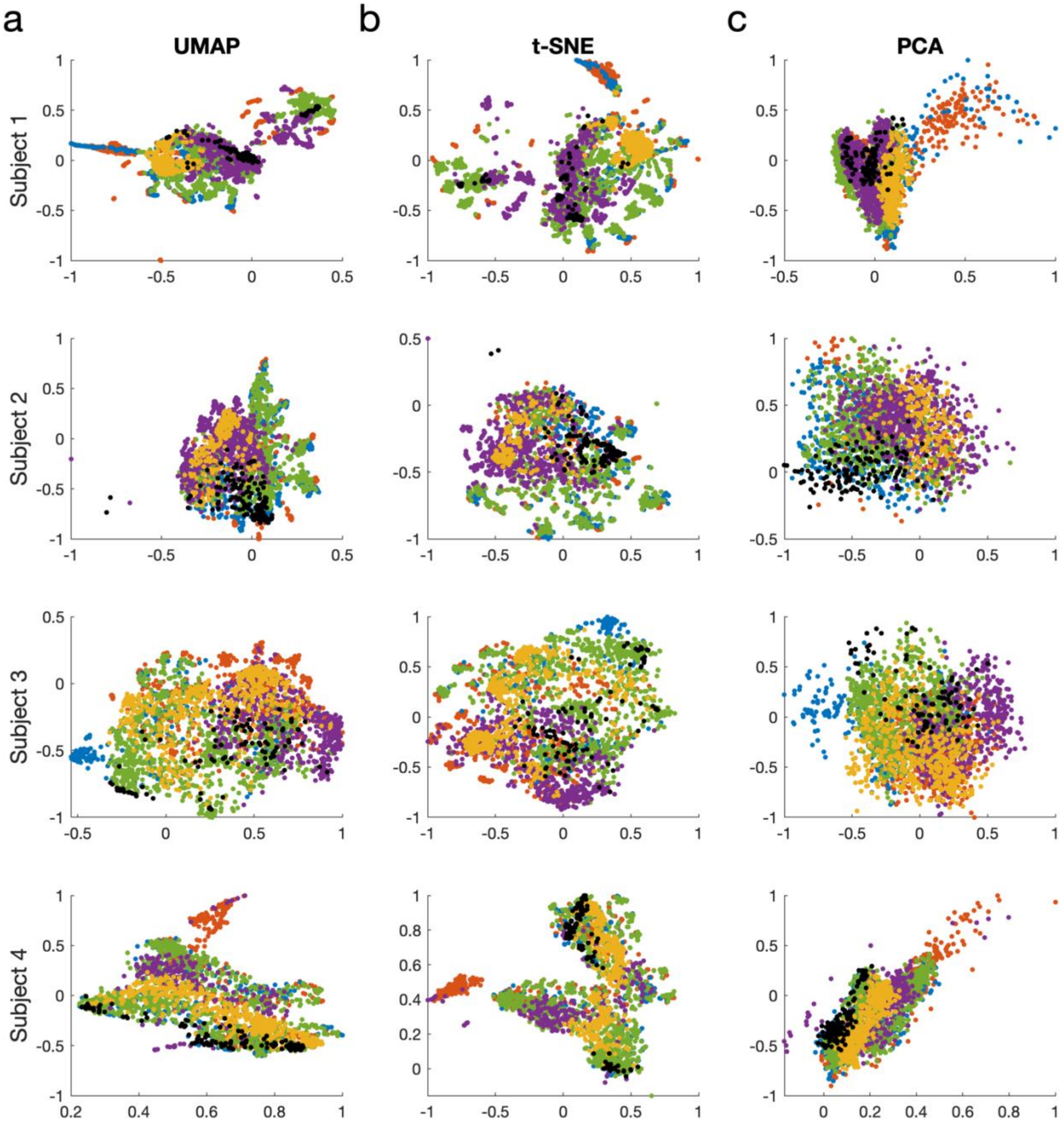

Figure S1: All subject-specific latent space representations.

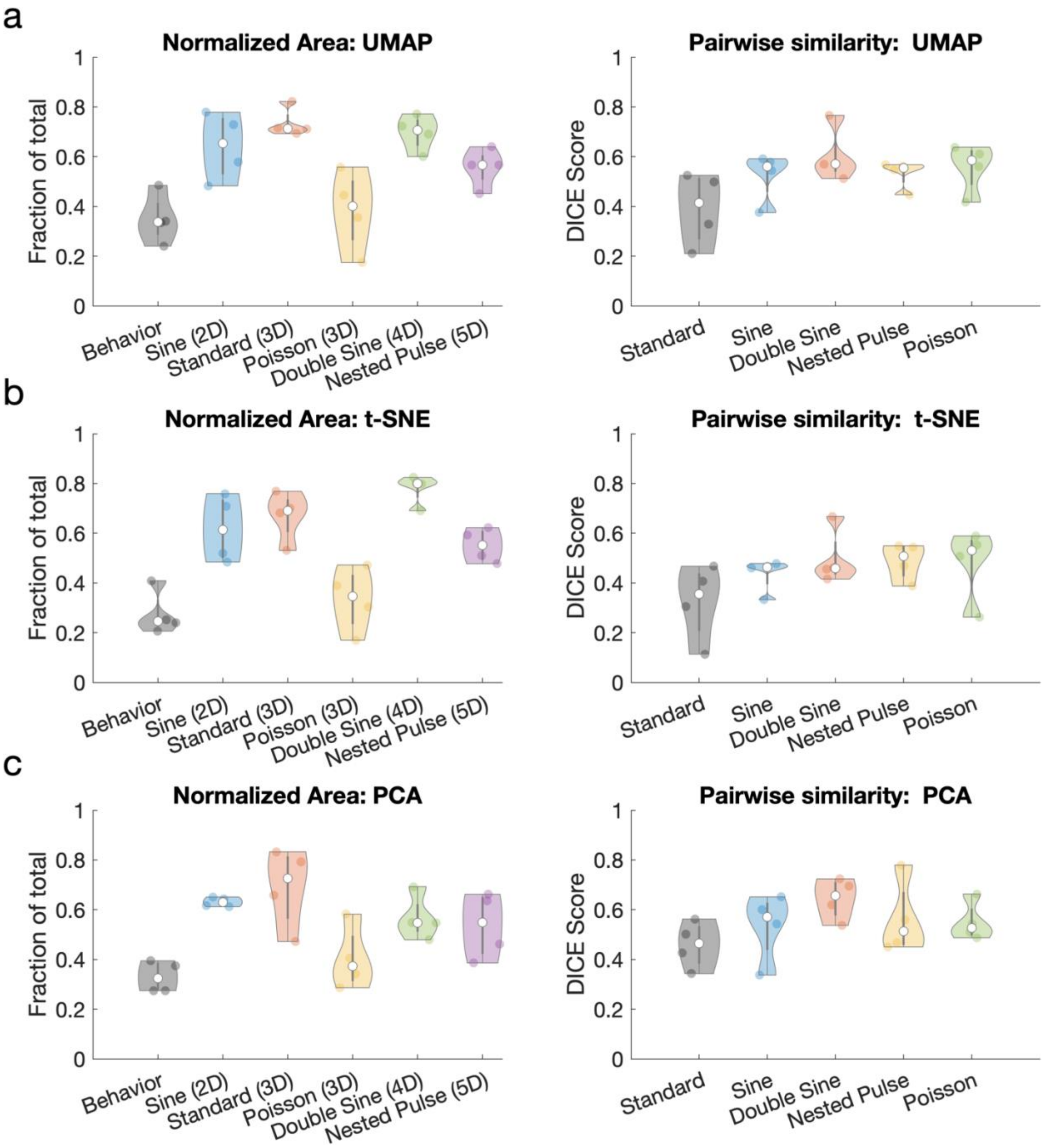

Figure S2: Figure panels 5c and 5f from the main text are replicated for all three dimensionality reduction algorithms used in the study (a: UMAP, as shown in figure 5; b: t-SNE; and c: PCA). Left: fraction of total area in the neural latent space. Right: Pairwise similarity vs. behavioral recording boundaries.

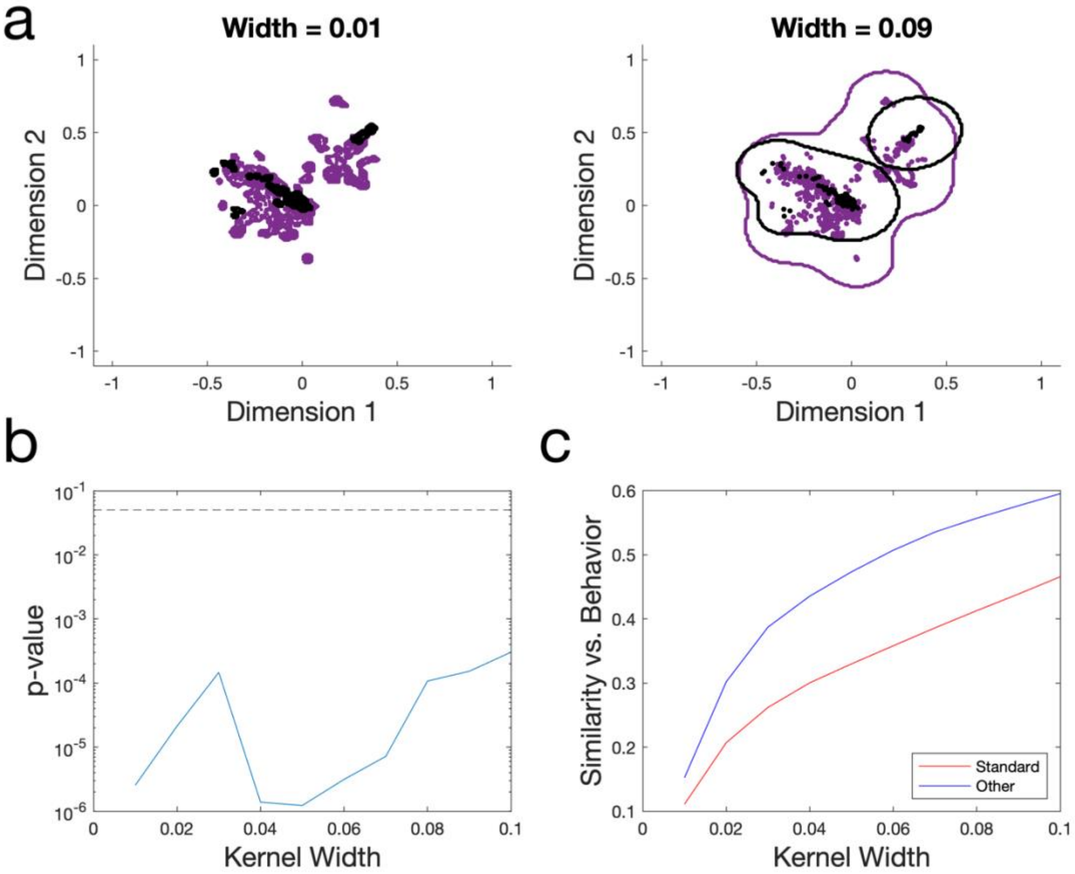

Figure S3: Behavioral similarity of stimulation waveforms vs. kernel width used for boundary determination. This figure demonstrates how increasing or decreasing the boundary size computed in the neural latent space influences the results demonstrated in Fig. 5f. a) Example boundaries, computed using the UMAP data shown in Fig. 5e, for low (left) and high (right) Gaussian kernel width values. b) Bootstrapped Friedman test p-value vs. Gaussian kernel width. c) Boundary overlap between stimulation waveforms and behavioral activity, shown for standard pulse stimulation (red) and averaged for all other stimulation waveforms, vs. Gaussian kernel width. The standard pulse waveform features reduced similarity to behavioral activity compared to other waveforms, consistent across all evaluated kernel width values.

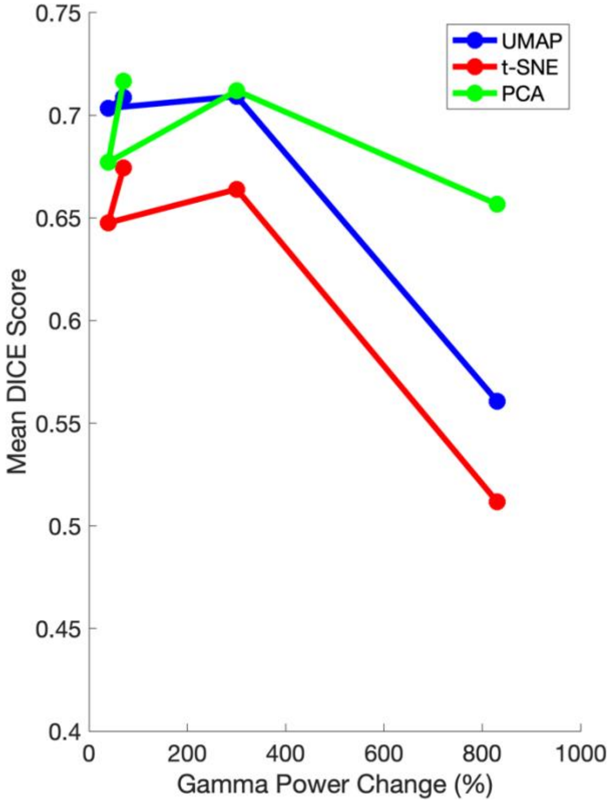

Figure S4: Optogenetic stimulation response magnitude predicts separability between neural latent space representations. Y-axis: Magnitude of the average overlap between all parameter space boundaries plotted separately for 4 subjects and 3 dimensionality reduction algorithms. X-axis: The magnitude of gamma power increase from baseline observed for low-amplitude (10 mW/mm^2), 35-Hz stimulation parameters. The negative trend in average overlap vs. optogenetic sensitivity may provide an explanation for the between-subject variability in the separability of the latent space representations: less clearly separable representations may result from weaker stimulation effects, whereas the most clearly separable representation was observed for the subject with the strongest response at low-amplitude parameters.
